# Selective Laser Melted Magnesium Alloys: Fabrication, Microstructure and Property

**DOI:** 10.3390/ma15207049

**Published:** 2022-10-11

**Authors:** Yun Zhou, Kai Zhang, Yaru Liang, Jun Cheng, Yilong Dai

**Affiliations:** 1 Department of Automotive Engineering, Hunan Industry Polytechnic, Changsha 410208, China; 2 School of Materials Science and Engineering, Xiangtan University, Xiangtan 411105, China; 3 Key Laboratory of Biomedical Metal Materials, Northwest Institute for Nonferrous Metal Research, Xi’an 710016, China

**Keywords:** selective laser melting, magnesium alloys, microstructure, mechanical property, corrosion behavior

## Abstract

As the lightest metal structural material, magnesium and its alloys have the characteristics of low density, high specific strength and good biocompatibility, which gives magnesium alloys broad application prospects in fields of biomedicine, transportation, and aerospace. Laser selective melting technology has the advantages of manufacturing complex structural parts, high precision and high degree of freedom. However, due to some disadvantages of magnesium alloy, such as low boiling point and high vapor pressure, the application of it in laser selective melting was relatively undeveloped compared with other alloys. In this paper, the fabrication, microstructure, mechanical performance and corrosion resistance property of magnesium alloys were summarized, and the potential applications and the development direction of selective laser melting magnesium alloys in the future are prospected.

## 1. Introduction

**Figure 1:**
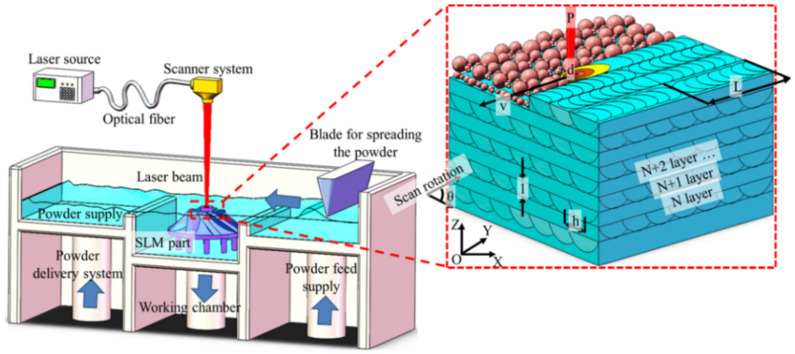
Schematic diagram of selective laser melting. Reprinted with permission from ref. [6]. Copyright 2020 Springer Nature.

With the advantages of low density, high specific strength, good thermal conductivity and good biocompatibility, magnesium and its alloys are the lightest metal structural materials in practical applications [1,2,3], which are applied in transportation, communication electronics, aerospace, biomedicine and other fields. However, the Mg alloy workpieces manufactured by traditional technology has some shortcomings, such as poor microstructure uniformity, long processing cycle, low material utilization and low efficiency [4]. Thus, the development and application of high-performance magnesium alloys were restricted. Selective laser melting (SLM) is a kind of additive manufacturing technology that uses high-energy laser beam to selectively sinter and stack powder layer by layer. It has the advantages of manufacturing complex structural parts, high precision, high degree of freedom and refining alloy structure [5]. Owing to that, the selective laser melting process has become an important method to prepare high-performance magnesium alloy workpieces. The principle of SLM technology is shown in Figure 1 [6]. According to the geometric data model in 3D software, the metal powder layer is selectively melted by a high-energy laser beam, forming a large number of molten pools. After that, the produced molten pool solidifies rapidly (103–106 k/s), and finally the parts with the required shape are obtained [7,8,9]. SLM technology has the following characteristics: (1) Suitable for a wide range of processing materials, including refractory metals, high reflectivity materials and low melting boiling point metals. (2) The forming accuracy is high. After grinding, sandblasting and other subsequent treatment, the surface of workpieces can meet the accuracy requirements. (3) It can process structures with complex shapes, such as spatial curved porous structure, light lattice sandwich structure, special-shaped complex cavity structure, etc., [10]. SLM technology is developed and widely used in iron [11], titanium [12,13], nickel base superalloys [14,15], aluminum alloys [16], and so on [17]. However, due to its active chemical properties, high affinity with oxygen, low melting boiling point and high vapor pressure, the application and development of magnesium alloys in SLM technology are relatively behind. In this review, the characteristics of the forming process of magnesium alloy by SLM technology were summarized, how parameters in the forming process affected the microstructure and properties of magnesium alloys reported in recent research were reviewed, and the possible applications of SLM forming magnesium alloys in the future are prospected.

## 2. Fabrication of Magnesium Alloy with Selective Laser Melting

### 2.1. Influence of Magnesium Alloy Powder

Since metal or alloy powders are used as raw materials in SLM process, the characteristics and quality of powders have a great impact on the stability of SLM process and the performance of final samples. The quality of powder is determined by its size, shape, surface morphology, composition and internal porosity. In this paper, several factors related to product performance will be reviewed.

#### 2.1.1. Alloying Elements of Mg in SLM

Due to the low melting point of magnesium (923 K), it is easy to burn in the SLM process. Thus, addition of alloy elements is beneficial to broaden the melting point and boiling point range of magnesium alloys, and further limit the selective vaporization of magnesium elements [18,19,20,21]. In addition, the research shows that the oxidation of magnesium oxide alloy powder can be prevented [22,23]. For example, in Mg–Be and Mg–Al alloys, active metal elements added to the alloy are required to react before Mg elements, so as to prevent large-scale oxidation of Mg elements [24,25,26,27]. Therefore, Mg Al, AZ (Mg–Al–Zn), ZK (Mg–Zn-Zr), AM (Mg–Al–Mn) and WE (Mg–Re) series are the most commonly used alloy systems in SLM process [23,28,29].

#### 2.1.2. Shape and Size of Alloy Powder

Spherical powder is usually used in SLM processing because it helps to improve the fluidity of the powder and obtain high precision. The research shows that because the irregular shaped powder is not easy to flow and has a strong tendency to aggregate, the use of non-spherical powder particles in SLM processing will make a negative impact on the uniform deposition of powder [30]. The shape of magnesium powder plays an important role in fluidity and laser reflection behavior [31]. Irregular shaped powder will significantly reduce the fluidity of the powder, cause uneven distribution of the powder and affect the quality after forming. Instead, the spherical powder with a uniform and smooth surface can improve the fluidity of the powder and the formability of magnesium alloy [32]. In particular, the powders should be without defects such as satellite powder and caking, which will lead to insufficiency fusion between the particles, thereby affecting the densification process [33].

**Figure 2:**
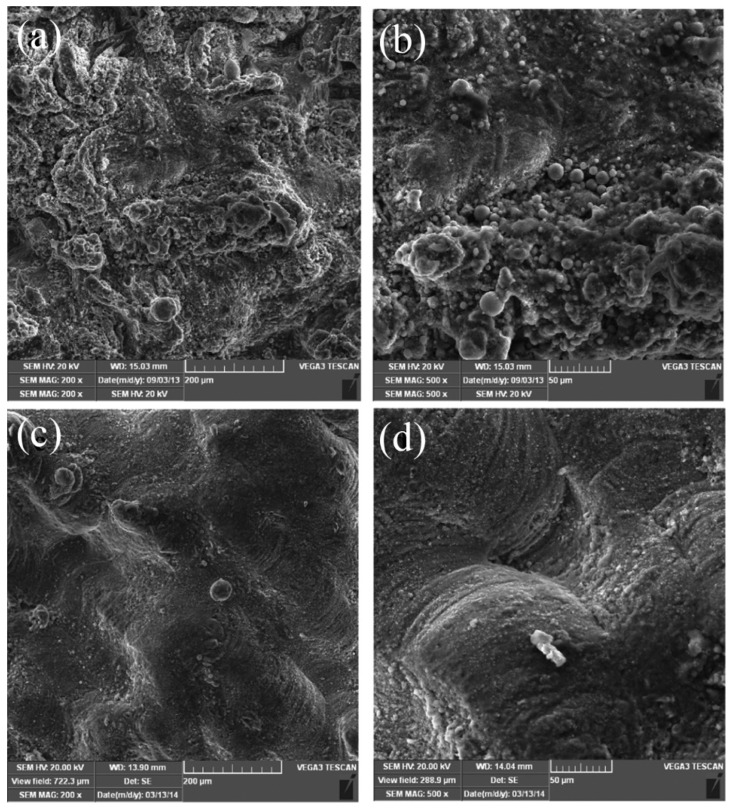
SEM topology under different magnifications of specimens fabricated using magnesium powders with granularity of (**a**,**b**) 400 mesh and (**c**,**d**) 250 mesh. Reprinted with permission from ref. [36]. Copyright 2015 Taylor and Francis Group.

The behavior of powders with different sizes is different in the laser processing process. SLMed workpieces that used powders with large particle size always showed poor adhesion between each layer and low density, due to the poor penetrating ability of it. Although relatively fine particles and high laser energy density can be used to prepare high-density components with better surface, fine powder particles are easy to be blown away by the protective air flow, thus affecting the deposition process. Furthermore, powder with small size will evaporate when comes to the situation of high laser power, the consequent generation of smoke and dust in the print bin will affect the output of laser energy and make the formed sample performed a poor surface [34]. In the recently work of Wang [35], the influence of powder particle size on smoke and dust generation in the process of forming AZ91D Magnesium Alloy by SLM was studied. The fine powders are easier to melt than the coarse powders, and more likely to experience the overburning phenomenon under the same laser energy density. Screening the fine powder below 20 µm can effectively reduce the amount of smoke and dust, and better surface quality and higher tensile strength can be obtained. Dong et al. [36] studied the size effect of Mg powder in SLM process. As shown in Figure 2, the sample fabricated by 400 mesh powder showed a rough surface with un-melted particles (Figure 2a,b), while the sample prepared with 250 mesh powder was well consolidated (Figure 2c,d). The reason for this result can be expressed as: with higher temperature and the bigger molten pool, some un-melted or melted powders were blown away by the air flow when fine powder particles were used. These powders adhered to the substrate and made 400 mesh powders exhibit more severe balling and agglomeration than that of 250 mesh powder. Aside from the size effect of powder, due to the different melt speed between larger size powders and smaller size powders, the uneven distribution of powder particles on the construction platform should be avoided. When the powders adopted show a wide size distribution mixed with both large-sized particles and small-sized particles, more attention should be paid to the selection of process parameters to avoid uneven melting of the powders. Spherical powders with a narrow range of particle sizes can help to improve the thermal conductivity of the powders, resulting in an increase in the density of SLM formed parts [37].

### 2.2. Influence of SLM Process Parameters

**Figure 3:**
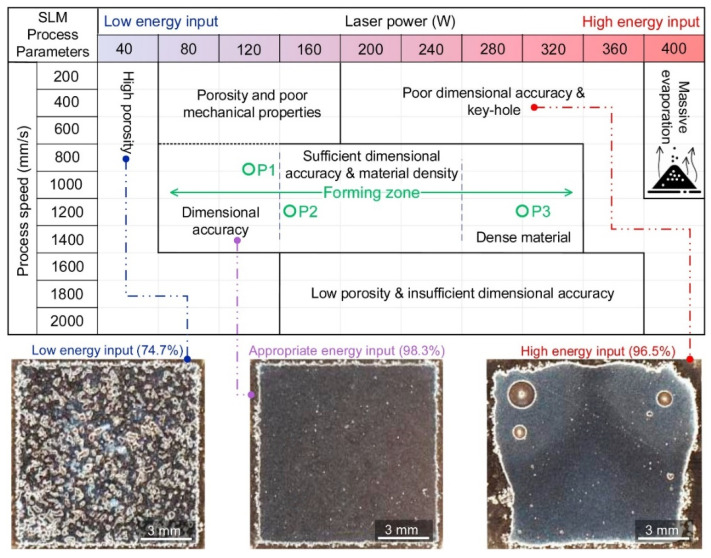
Effect of SLM process parameters on the formability of WE43. Reprinted with permission from ref. [40]. Copyright 2020 Elsevier.

The main advantage of SLM technology is that it can produce metal parts with high density. However, since the absence of mechanical pressure, fluid dynamics is mainly driven by gravity, capillary action and thermal effect, so it is not easy to achieve this goal. In addition, the lack of mechanical pressure during the SLM process may reduce the solubility of some elements during solidification, resulting in discontinuous melting to form pores and surface irregularities. Therefore, appropriate parameters (include power density, scanning distance and speed, and layer thickness etc.) need to be carefully selected to obtain parts with good quality. Taking WE43 as an example, Figure 3 shows the influence of SLM process parameters on the formability of magnesium alloy. When specimens were produced in the low energy input zone, they exhibited poor mechanical strength due to the extensive presence of process-induced pores and lack of fusion. At high energy inputs, a loss of the Mg content occurred due to its low boiling point. An appropriate volume energy density (*E_V_*) can be obtained by adjusting process parameters [38]. Volume energy density *E_V_* is defined as the laser energy per volume and can be calculated by the following equation:

(1) $$E_{V}=P/vdh$$

where *P* (W) is the laser power, *V* (mm/s) is the scanning speed, *D* (mm) is the scanning distance, *h* (mm) is the layer thickness. Under proper laser parameters, the SLM prepared can achieve a negligible fraction of metallurgical and process-induced defects. However, in the recent work of Deng [39], the depth and width of molten pool and the porosity vary significantly despite using the same LED value but different combinations of *p* and V values. When *p* or V is changed alone, it is reliable to use LED as a design parameter, but when *p* and V are changed at the same time, LED has limitations.

#### 2.2.1. Laser Energy Density

**Figure 4:**
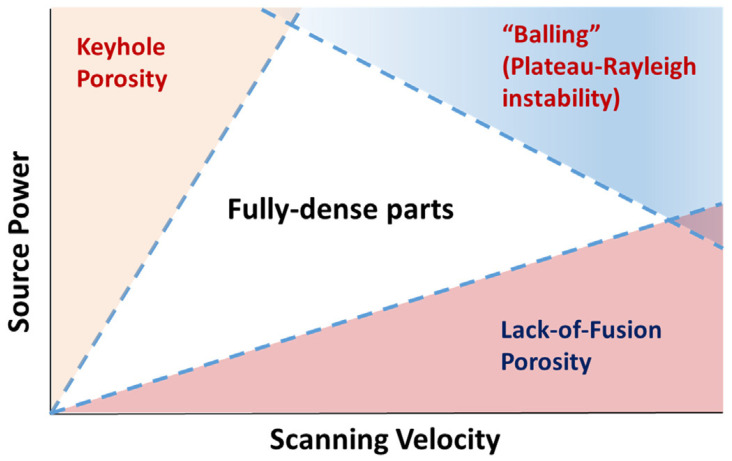
Illustration of processing parameter influence on porosity. Reprinted with permission from ref. [17]. Copyright 2020 Elsevier.

In the SLM forming process, the densification process of the sample is as follows: first, the metal powder is melted by a single laser beam, and then its melting trajectory is overlapped with the adjacent melting trajectory. When the multilayer is formed, the laser beam irradiates the powder layer to melt it and weld it with the previous powder layer to form a solid interlayer bond. Olakanmi et al. [41] observed that under the appropriate laser power and scanning speed, the metal powder can be completely melted, and there is good solid–liquid interface wettability between the powder particles and the melt, so that the formed sample can be nearly completely dense. The complete melting of metal powder can not only enhance the adhesion between powders and form complex structural parts, but also facilitate the discharge of gas, reduce the formation of pores in the sample, obtain more dense formed parts and obtain excellent comprehensive properties. Shown in Figure 4, the processing parameter influence on porosity was summarized in Oliveira’s work [17]. Low *E_V_* is not enough to melt magnesium alloy powder completely, and it is difficult to obtain dense formed parts. Too high *E_V_* will cause surface spheroidization and burning of alloy elements [42]. For very high energy densities, Mg vapor will generate back pressure on the molten pool, and the huge temperature gradient of the molten pool causes a strong Marangoni convection effect in the molten pool, and the depth of the molten pool is much greater than the width of the molten pool. However, the solidification rate of SLM process is very fast, and more Mg vapor has no time to escape from the molten pool, so it exists in the molten pool in the form of pores, which will make the workpiece more porous. Thus, a depression filled by steam and external gas was formed, and its morphology is called keyhole, which may degrade the fatigue life of the part by acting as a crack initiator [43,44]. Increasing the canning speed is a simple way to reduce the formation of keyholes, but at high energy density, too large scanning speed will also bring about “balling”. In order to address defects such as keyhole porosity, lack-of-fusion porosity, and “balling”, suitable laser power and scanning speed need to be investigated.

**Figure 5:**
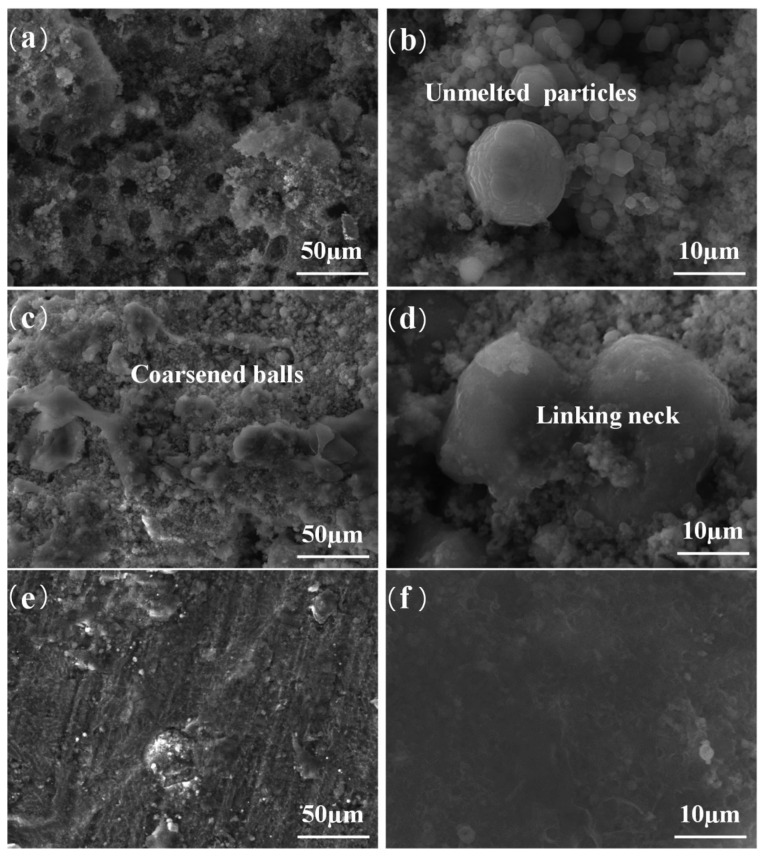
Surface of SLM tracks at various laser energy density: (**a**) 2.0 J/mm, (**c**) 5.0 J/mm, (**e**) 10.0 J/mm, (**b**,**d**,**f**) are high resolution of (**a**,**c**,**e**), respectively. Reprinted with permission from ref. [48]. Copyright 2016 Taylor and Francis Group.

In the study of Wang with Mg–Y–Sm–Zn–Zr alloy [45], it was found that the size of molten pool was positively correlated with laser power. As the laser power increased from 40 W to 80 W, the length and depth increased from 71.14 μm and 21.62 μm to 110.8 μm and 31.9 μm, respectively. Zhang et al. [46] studied the effect of laser energy density on the density of Mg–9%Al alloy samples by adjusting laser power and laser scanning speed. The results show that at a low laser energy density, the powder melts incompletely, resulting in discontinuous scanning tracks and spheroidization, which leads to more pore defects in the sample. With the increase of laser energy density, the powder melts better, so that more liquid phase can flow and penetrate into the gap between particles. The relative density of the sample is higher, and the surface is relatively smooth. However, with the further increase of laser energy density, the powder will melt completely, and defects such as spheroidization and scum will appear in the molten pool, resulting in reduced density and poor surface finish. Wei et al. [47] observed the formability of AZ91D alloy under different bulk energy densities. When the bulk energy density is 83–167 J/mm^3^, the sample has no obvious macroscopic defects and has a high density. When the bulk energy density is higher than 214 J/mm^3^, the alloy elements volatilize and burn seriously, and the sample cannot be deposited and formed. In Yang‘s research [48], the SLM printing process shows that with a low laser energy density, the powders were in a discrete state, and there was no fusion between the powders (Figure 5a). By increasing the laser energy density, the powders can be partially melted and sintered together forming sintering neck as a weak bonding (Figure 5d). It is only when the laser density is large enough to melt the powder completely that smooth and continuous trajectory can be obtained, as shown in Figure 5f. With the further increase of laser energy density, the powder evaporates. In Ng’s study of magnesium [49], the laser energy density also shown an effect on the average grain size of α-Mg grown. It changed from 2.30 μm to 4.87 μm with the laser energy density increasing from 1.27 × 10^9^ J/mm^2^ to 7.84 × 10^9^ J/mm^2^. It is attributed to the high energy density which keeps the Mg-melt at a relative high temperature state, affected the cooling process of the melt and resulted in grain growth.

Although the SLM process is usually based on the complete melting, partial melting of powder caused by low energy density is not necessarily bad. The molten metal on the surface of partially melted particles can make the powders adhere to each other and leave pores between the powders, which can be effectively used to produce porous structures with complex shapes [50], whereas a loose structure formed at energy inputs below 77 J/mm^3^ due to “balling effect” and incomplete melt of powders. It is worth noting that the specific value of energy density is meaningful only in specific cases, because different overlapping distance and powder layer thickness will affect the energy density, and the same energy density does not necessarily ensure the stability of the quality of the formed sample [51], so the best E_V_ value depends on the specific powder material composition and laser beam scanning forming strategy [52].

#### 2.2.2. Scanning Speed and Spacing

**Figure 6:**
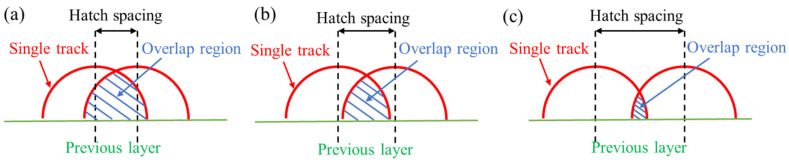
Schematic diagram illustrating the effect of hatch spacing on area of overlap region: (**a**) low hatch spacing; (**b**) suitable hatch spacing; (**c**) high hatch spacing. Reprinted with permission from ref. [43]. Copyright 2020 Elsevier.

At a constant laser power, the scanning speed is related to the residence time of the laser beam on the surface of the molten pool. Thereby, by reducing the scanning speed, the energy density will be increased, and higher workpiece density will be obtained. The scanning distance (also known as the spacing distance) is another important parameter that affects the relative density of the alloy. It determines the degree of overlap of laser points when a new laser line sweeps through the previously scanned line. Ng et al. [53] studied the single pass experiment of magnesium alloy at different scanning speeds, and proved the feasibility of magnesium alloy powder in SLM manufacturing. With too fast scanning speed, powder splashing can be observed, which will cause instability of molten pool and increase thermal stress of the production. On the contrary, when the scanning speed is too low, it will lead to the recrystallization of magnesium alloy samples during solidification, which will affect the microstructure and morphology of the alloy. In Wei’s work [47], the effects of scanning speed and scanning distance on the relative density of AZ91D alloy prepared by SLM were summarized. The relative density of the sample decreases with the increase of scanning speed and hatch spacings. Similar results were also obtained for ZK60 alloy [54]. As the scanning speed increased, the relative density of the sample reached a peak of 94.05% at 300 mm/s. When the scanning speed was 100 mm/s, serious vaporization and metal powder burning were observed, leaving ablative pits on the surface of the substrate, resulting in the termination of the molding process. When the scanning speed is higher than 500 mm/s, the powder particles cannot be completely melted, and pores will be formed between unmelted powders, resulting in a sharp decline in the relative density of the sample. The influence of scanning spacing on the overlap area between adjacent scanning passes was studied by Deng [43]. It can be seen from Figure 6 that suitable hatch spacing will make the height of the overlap region and the height of the unlapped region tend to be uniform, which will result in even powder distribution in the next layer. With a width of the molten pool approximately 140 to 200 μm, the corresponding overlap ratio is about 30–50% when the hatch spacing is 100 μm.

#### 2.2.3. Thickness of Powder Layer

The thickness of powder layer is another important parameter which has an important impact on porosity and interlayer adhesion, and also affects the tensile strength, hardness and dimensional accuracy in the construction direction. A thicker powder layer will reduce the thermal penetration depth of the laser beam, so the bottom powder cannot be effectively melted, and the adhesion of each layer is reduced, resulting in local stress concentration, uneven chemical composition, pores, microcracks and incomplete fusion areas. These defects greatly reduce the fracture toughness and tensile ductility of the alloy. A thicker powder layer will lead to non-fusion between particles, and the same amount of laser energy will radiate more powder than a thin powder layer. The laser energy density penetrating the powder bed will not be enough to completely melt the powder particles, which will cause more pores and reduce the density of the sample. Therefore, it is necessary to establish the best layer thickness parameters, so that there is a good adhesion between layers, and reduce the defects that may occur in the SLM process, so as to obtain a higher density. Savalani et al. [55] studied the influence of powder layer thickness on single channel pure magnesium prepared by SLM. In their experiment. The layer thickness of preheated samples was adjusted from 150 to 300 μm. The results show that there is a critical value (250 μm) for the thickness of the powder layer. When the powder thickness was relatively thin, a flat surface without any surface defects could be obtained. Because the amount of material to be melted is significantly less, the heat transmitted in the molten pool has enough energy to completely melt the adjacent particles, rather than partially melt.

## 3. Microstructure of SLMed Samples

**Figure 7:**
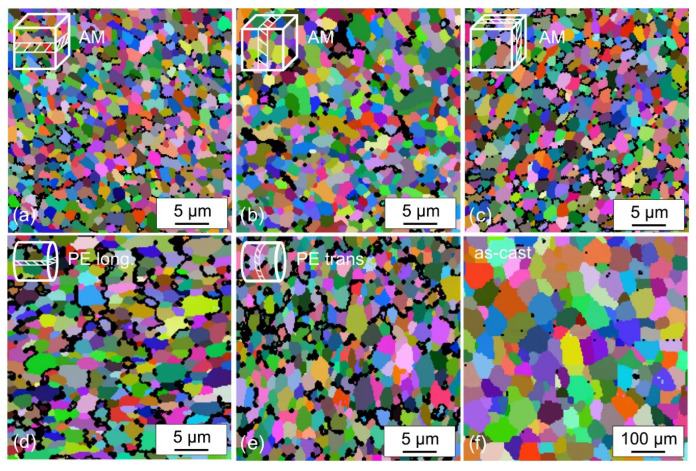
Electron backscatter diffraction of (**a**–**c**) selective laser melting, (**d**,**e**) of powder extruded, (**f**) of as-cast material. Reprinted with permission from ref. [58]. Copyright 2019 Elsevier.

When the incident laser beam irradiates the metal powder layer, most of the laser energy is absorbed by the metal powder particles, resulting in rapid heating and local melting of the powder. Therefore, laser selective melting process is a process of rapid melting and solidification [56]. Thus, by adjusting the process parameters and changing the thermodynamics and dynamics of the molten pool, so as to control the size and shape of grains, as well as the content and composition of phases in the solidification process, so as to obtain the required microstructure [57]. The characteristics of SLM with high cooling rate can obtain fine microstructure, while the cooling rate of Mg alloy can reach 10^6^ K/s during SLM. Compared with the traditional process, SLMed magnesium alloy is also affected by recrystallization, but it is easier to control the evolution of microstructure. In the research of Zumdick, as shown in Figure 7, due to the characteristics of powder sintering and rapid cooling in the SLM process, the magnesium alloy presents a uniform and fine microstructure (Figure 7a–c); Although the microstructure of magnesium alloy prepared by extrusion is relatively uniform and fine, it has serious anisotropy (Figure 7d,e); The microstructure grain of as cast state is relatively rough (Figure 7f) [58].

**Figure 8:**
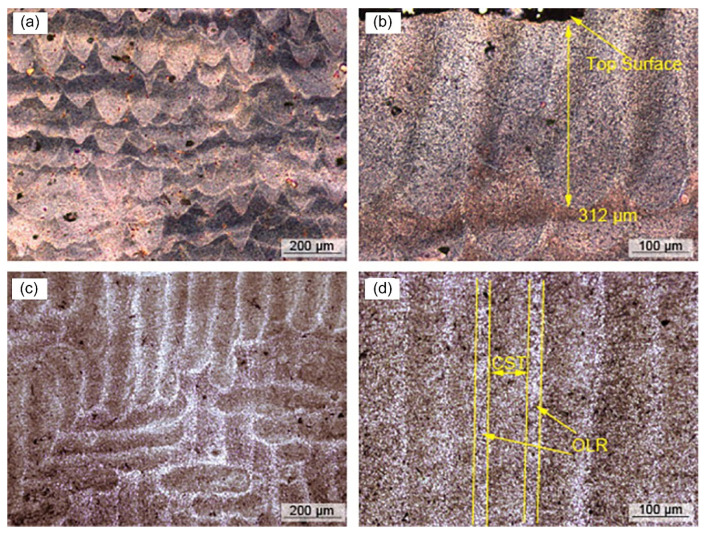
Optical micrographs of the AZ91D sample deposited at Ev of 166.7 J/mm^3^. (**a**,**b**) The vertical section, (**c**) the cross section, and (**d**) the detail of scanning tracks shown in (**c**). Reprinted with permission from ref. [47]. Copyright 2014 Elsevier.

The multiple remelting in the SLM process makes the microstructure change in different height directions, which is attributed to the different heat treatment, conduction, convection and radiation conditions between different layers. After repeated remelting, the cooling rate decreases relatively, which coarsens the grains in the remelted part. The microstructure is different due to the difference of heat affected zone between the edge and center of molten pool. In the preparation of AZ91D alloy by SLM, the grains in the scanning track area are smaller than those in the overlapping area, which is caused by the repeated remelting of the grains at the edge of the molten pool, the reduction of the cooling rate, and the existence of temperature gradient difference. Figure 8 shows the distribution of molten pool under the optical microscope of AZ91D sample under the bulk energy density of 166.7 J/mm^3^, which obeys the Gaussian distribution of laser energy. The molten pool with elliptical bottom is arranged layer by layer, which is the inherent layered feature of SLM technology. The molten pool is closely stacked, forming a good metallurgical bond between two adjacent layers. [47] In addition, due to the temperature gradient difference between the edge and center of the molten pool, the transformation from columnar crystal to equiaxed crystal may occur from the edge of the molten pool to the center. Columnar α-Mg grains occupy the edge area of the molten pool, while the α-Mg grains in the center area of the molten pool show equiaxed crystal state.

**Figure 9:**
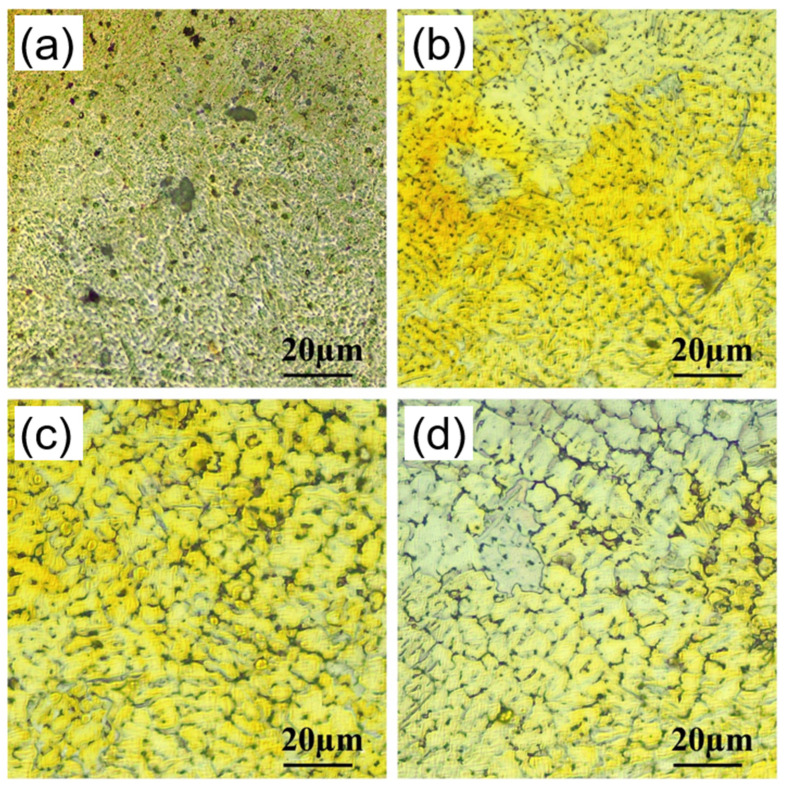
Microstructures of SLM ZK60 samples at different parameters, (**a**) 420 J/mm^3^; (**b**) 500 J/mm^3^; (**c**) 600 J/mm^3^; (**d**) 750 J/mm^3^. Reprinted with permission from ref. [59]. Copyright 2017 Elsevier.

The microstructure of SLM samples is closely related to the preparation parameters. In the study of Mg–Zn–Zr (ZK60) alloy (Figure 9). It is found that, with the increase of laser energy density, the crystal structure changes into: columnar crystal, equiaxed crystal, and coarsened equiaxed crystal, which is caused by the increase of laser energy density and the decrease of its cooling rate [59]. In addition to the adjustment of preparation parameters, appropriate component selection and subsequent processing can effectively control the microstructure of samples. SLM will lead to changes in composition and microstructure, which is due to the high vapor pressure of Mg, Zn and other elements. During the printing process, Mg, Zn and other elements will be selectively evaporated, and Al, Zr and other elements will be enriched on the surface through solute capture effect. In the process of rapid laser melting, due to the great temperature gradient difference, it is conducive to the formation of Marangoni convection and promotes the uniform dispersion of alloy elements in the molten pool. The expansion of the solid–liquid interface contributes to the solute capture phenomenon in the α-Mg matrix, and more solute atoms are concentrated in the α-Mg matrix, which improves the solid solution limit of alloy elements in the α-Mg matrix and inhibits the nucleation of β- phase [47,59]. Therefore, the change of composition will affect the microstructure during laser selective melting. In order to obtain finer microstructure, severe plastic deformation is a very effective processing method. After extrusion and heat treatment, the mechanical properties of JDBM alloy are improved at room temperature compared with those as cast [60]. The main reason is that after extrusion, the process will undergo dynamic recrystallization, resulting in grain refinement.

## 4. Properties

### 4.1. Mechanical Property

#### 4.1.1. Hardness

The rapid solidification effect on the parts in the SLM process leads to the grain refinement of its microstructure, which is one of the main reasons for the increase of the hardness of SLM manufactured parts. Thus, the hardness performance of samples can effectively be controlled by adjusting the processing parameters in SLM process. High cooling rate stimulated by low laser energy density can refine the grain size, consequently, increase the hardness values in the melted zone. In addition, according to the solid solution strengthening theory, high solid solubility can also improve the hardness of products [61]. Therefore, the solid solution of different elements also affects the hardness value of magnesium alloy. For example, the microhardness of Mg–Al alloy is different from that of α-Mg. In the study of Cáceres, the hardness was increasing with the aluminum content between 1%~8% in Mg–Al alloys [62]. In Table 1, the relationship between hardness values of various magnesium alloys was summarized. It can be seen from the table, beside the grain size, the hardness value of SMLed magnesium alloy is closely related to the microstructure, element composition and content. Moreover, according to Mercelis and Kruth [63], the residual stress maintained at a reasonable level in the parts manufactured by SLM can improve its hardness, provided that sufficient densification can be achieved without cracks or pores.

#### 4.1.2. Tensile Properties

**Figure 10:**
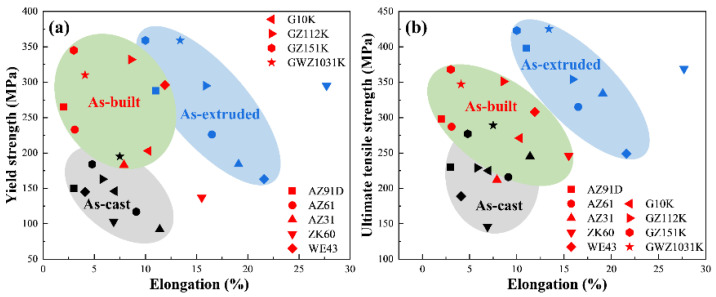
Comparison of room temperature tensile properties of the typical Mg alloys under the as-cast, as-built and as-extruded states (**a**) YS vs. EL, (**b**) UTS vs. EL. Reprinted with permission from ref. [74]. Copyright 2022 Chinese Academy of Sciences.

Previous studies have shown that the elastic modulus of SLMed magnesium is related to powder thickness and energy density. In Ng’s study [73], pure Mg prepared with energy density of 7.84 × 10^3^ J/mm^2^ can reach an elastic modulus of 32.83–34.97 GPa. which are higher than the conventional cast magnesium (∼28 GPa). Wei et al. [47] studied the tensile properties under different volume energy densities. The results showed that, at a relatively low energy density, the ultimate tensile strength of the specimen with an energy density of 83.3 J/mm^3^ was 274 MPa. The solid solubility of Mg in the sample was restricted, and the precipitation of the second phase was also relatively reduced, which further affects the tensile properties of SLM formed samples. With an increased energy density of 166.7 J/mm^3^, the ultimate tensile strength of the specimen reached 296 MPa, which is superior to the die-cast AZ91D (~230 MPa). In the study of Savalani [55], the powder layer thickness also affected the SLM of magnesium. When the thicknesses of the powder layers were set to 0.15–0.20 mm and 0.25–0.30 mm, the elastic moduli of the samples were 31.88–34.28 GPA and 28.43–31.47 GPA, respectively. In the work of Peng [74], the room temperature tensile properties of typical magnesium alloys in as cast, SLM and extruded states were compared. As shown as Figure 10, the yield strength of SLM magnesium alloy is significantly higher than that of as cast alloy, close to or even higher than that of extruded alloy, which is mainly due to the fine grains in SLM state. The tensile strength of SLM magnesium alloy is mostly significantly higher than that of as cast alloy and lower than that of extruded alloy. The elongation of SLM magnesium alloy is generally low, and it does not show the trend of improvement of the plasticity which may be caused by fine grains. For example, the elongation of AZ91D alloy in SLM state is 1.24–1.83% with the laser energy density of 83.3–167.7 J/mm^3^, which is lower than the elongation of die-cast AZ91D (3%) [47]. Similarly, due to the β-Mg_17_Al_12_ phase precipitated along the grain boundaries, the elongation of SLMed AZ61 alloy with the laser energy density of 138.89–208.33 J/mm^3^ is 2.14–3.28%, which is lower than the elongation of 5.2% of AZ61 as cast [75].

#### 4.1.3. Fracture Behavior

**Figure 11:**
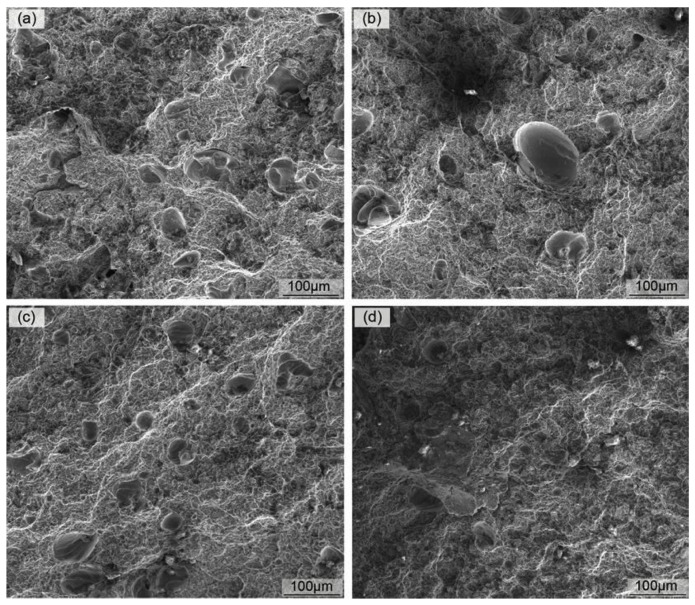
Fracture topography of SLMed AZ91D at different energy densities (**a**) 166.7 J/mm^3^, (**b**) 142.9 J/mm^3^, (**c**) 111.1 J/mm^3^, (**d**) 83.3 J/mm^3^. Reprinted with permission from ref. [47]. Copyright 2022 Copyright 2014 Elsevier.

The fracture behavior analysis of SLM parts shows the characteristics of ductile brittle hybrid fracture. Wei et al. [47] analyzed the fracture morphology of SLMed AZ91D alloy (Figure 11). All tensile specimens showed ductile brittle fracture characteristics. This fracture mode is mainly related to the mixing of soft α-Mg and brittle hard eutectic multiphase structure. It can be seen from Figure 11 that the dimple of the alloy is shallow, and there are defects such as pores and microcracks on the micro fracture diagram, resulting in poor plasticity of SLMed alloy. In addition, due to the layer-by-layer manufacturing method, it is observed that the construction direction of the part during SLM will affect the final tensile properties of the part. Samples deposited along the length direction of the tensile sample usually show higher tensile strength than samples deposited perpendicular to its length. Performing hot isostatic pressing (HIP) procedures after SLM can significantly reduce the anisotropic mechanical behavior of the parts by reducing manufacturing-induced pores [76]. However, the elongation at break of SLM parts is usually low, which may be attributed to micropores and oxide inclusions in the parts, which is the result of non-optimized SLM process. It is worth noting that, even at room temperature, magnesium can be oxidized under extremely low oxygen partial pressure [77,78], and local oxidation caused by residual air in the powder gap can often be observed in SLMed magnesium alloys [79,80]. Oxide films are believed to inhibit the densification mechanism and induce spheroidization, and then destroy the intergranular coalescence/wetting between the molten layers. Oxide inclusions, which usually lead to crack initiation and reduce the mechanical properties of SLM manufactured parts [81,82], need to be avoided in research.

#### 4.1.4. Effect of Heat Treatments on the Mechanical Properties

Although the cooling rate of SLM process is very high, most of the SLM magnesium alloys still have hard brittle eutectic phase on the grain boundary and cannot form single-phase supersaturated solid solution. Therefore, it is necessary to carry out subsequent heat treatment to adjust the microstructure and improve the mechanical properties especially for the tensile properties. Room temperature tensile properties of some magnesium alloys under the state of SLMed and post-treatmented were summarized in Table 2.

**Figure 12:**
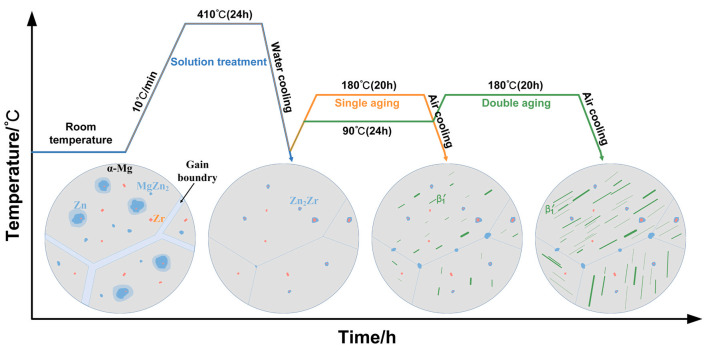
Schematic illustration of the microstructure evolution of LPBF ZK60 Mg alloy during different heat treatments. Reprinted with permission from ref. [83]. Copyright 2022 Elsevier.

Hyer et al. [84] conducted a heat treatment of solutionizing at 536 °C for 24 h and subsequent ageing at 205 °C for 48 h on SLM state WE43 alloy. High temperature and long-time solid solution treatment led to significant coarsening of grain. Meanwhile, the β_1_-Mg_3_Nd phase was found to dissolve, and developed into plate-like precipitates. The combined action of the two causes made the tensile strength remain unchanged at 251 MPa, and the yield strength slightly increase from 215 MPa to 219 MPa, and the elongation to increase from 2.6% to 4.3%. The above T6 heat treatment has not significantly improved the mechanical properties, so it is necessary to develop a special follow-up heat treatment system for the unique rapid solidification non-equilibrium structure in SLM state. In Fu’s study of GZ151K [86], after subsequent aging at 200 °C for 64 h (T5 treatment), both the strength and elongation of the SLMed-T5 GZ151K increased. The YS, UTS and elongation of the SLMed-T5 GZ151K alloy are 410 MPa, 428 MPa and 3.4%, respectively, which are higher than those of the as-fabricated (SLMed) GZ151K alloy (YS of 345 MPa, UTS of 368 MPa and elongation of 3.0%) and conventional cast-T6 alloy (YS of 288 MPa, UTS of 405 MPa and elongation of 2.9% [88]). The performance improvement brought by the subsequent aging was attributed to the YS improved by precipitation hardening and the improvement of ductility due to the probably released part of the residual stress. Recently, Liang et al. [83] studied the evolution of microstructure and mechanical properties of SLM ZK60 alloy under different heat treatment systems. As shown in Figure 12, the crystal boundary of SLM ZK60 magnesium alloy is composed of reticulated Mg_7_Zn_3_ phase, and there are three precipitates (Zn rich phase with Zr rich core, Zn rich phase without Zr and Zr rich particles) in the crystal. After the solution heat treatment (410 °C for 24 h), the Mg_7_Zn_3_ phase at the grain boundary is dissolved into the matrix, and the precipitated phase in the crystal mainly evolves into the Zn rich phase with Zn_2_Zr core. The T4 heat treatment reduces the strength and slightly increases the elongation from 15.5% (SLM state) to 16.7%; Rod-like precipitates after aging heat treatment β_1_’- MgZn precipitates can significantly improve the mechanical properties. The length and quantity of the rod-shaped precipitates of the two-stage aging (90 °C for 24 h + 180 °C for20 h) are more than that of the single-stage aging (180 °C for 20 h). Compared with the single-stage aging, the strength of the two-stage aging can be improved while maintaining a considerable plasticity. T6 heat treatment can significantly improve the yield strength and slightly reduce the elongation to 14.1%.

#### 4.1.5. Abrasion Resistance

Chang et al. [89] used additive manufacturing equipment to prepare Mg–Zn–Ca alloy, and explored the Mg–Zn–Ca alloy at wear behavior in 3.5 wt.% NaCl solution. The dry friction and wear mechanism of the alloy is adhesive wear and oxidation wear, while the main wear mechanism of wet friction is corrosion and adhesive wear. When the sliding distance is 28 m, the wet friction process first enters the stable wear stage compared with the dry friction process. The average wear width of wet friction is larger than that of dry friction, and the average wear rate of wet friction is more serious than that of dry friction.

### 4.2. Corrosion Resistance Properties

Generally, poor corrosion resistance of magnesium alloys caused by high chemical activity of magnesium alloys and the lack of protective passivation oxide film was considered as a major obstacle to its further application [90,91]. In addition, with a high negative standard electrode potential, magnesium and its alloys always showed a rapid corrosion feature under similar body fluid conditions [92]. The hydrogen produced by corrosion cannot be treated by the host tissue, which have prevented magnesium-based materials from being applied in practice so far. Therefore, the corrosion rate of magnesium and its alloys should be carefully controlled. The corrosion properties of magnesium alloys are closely related to their phase and alloy elements. Biodegradable Mg alloys, such as Mg–Ca AZ31, AZ91, and WE43 have been reported with improved corrosion resistance [72]. The corrosion rates of different Mg alloy fabricated by SLM technology are summarized in Table 3.

Shuai et al. [71] found that the adding of zinc can alleviate the degradation rate of Mg–Sn alloy and improve the corrosion resistance by formation of Zn(OH)_2_ protective layer during the degradation process. However, with the further increase of Zn content, the content of Mg_7_Zn_3_ in the second phase increases, and galvanic corrosion occurs between the second phase and the matrix, which will accelerate the degradation of magnesium alloy. The research of Zhou et al. [95] shows that the addition of a certain amount of Sn can alleviate the corrosion of magnesium alloys. With the increase of Sn element, the second phase Mg_2_Sn will also increase and accelerate the degradation rate due to its galvanic corrosion. Therefore, adding a certain amount of Sn element will reduce the degradation rate of magnesium alloy. Similarly, ZK60–0.4Cu [71] alloy showed certain anti-degradation ability and strong antibacterial ability caused by alkaline environment and copper ions. He et al. [65] formed AZ61 alloy by SLM technology. The reduced the formation of β-Mg_17_Al_12_ phase can reduced the galvanic corrosion between α-Mg and β-Mg_17_Al_12_ phase and alleviated the corrosion of magnesium alloy to a certain extent. In Yin’s study of ZK30 [96], the corrosion degradation rate of the composites decreased by adding bioactive amorphous bioactive glass. Furthermore, the evenly distributed Mg_7_Zn_3_ phase was refined and acts as a barrier to slow down the corrosion rate. By adding 2 wt% Mn during the SLM processing of Mg–Mn alloy, Yang et al. [93] observed that the corrosion resistance of pure magnesium can be improved. It is attributed to the increase of corrosion potential and grain refinement caused by manganese solid solution.

Besides the factors mentioned above, grain refinement and uniform structure can also improve the corrosion resistance of the material [59]. In the study of AZ61 alloy [65], the formation of uniform and fine equiaxed grains improves its corrosion resistance. Due to the existence of more grain boundary areas in fine grains, the corroded α-Mg grain boundary can act as a certain corrosion barrier. In addition, fine grains can form a denser surface oxide film to prevent the reaction between Cl^−^ and Mg^2+^ ions in body fluid. In the study of the corrosion resistance of Mg–Nb–Zn–Zr (JDBM) alloy, Zhang et al. [97] used reciprocating extrusion technology to process JDBM alloy and found that its grain size was reduced to 1 μm, and its corrosion resistance has also been improved.

## 5. Potential Applications

Magnesium alloys have elastic modulus similar to human bones (2–30 GPA) [98], and Mg (Mg^2+^) is an essential element and regulatory ion in human body. Therefore, magnesium alloys have great application potential in biomedical materials [99,100]. Selective laser melting has a significant advantage in manufacturing medical porous metal structures, which can produce fine and porous structures while adapting to various shapes. This makes it the preferred technology for the production of metal stents and implants [101,102]. Many engineering materials have been used to produce complex porous/cellular structures through SLM technology. So, it is possible to produce porous structures with magnesium alloys. Li et al. [103] produced biodegradable porous magnesium, which is likely to meet all the functional requirements of an ideal bone substitute material. First of all, its mechanical performance is high enough to be used as a mechanical support. Secondly, the manufactured parts showed a fully interconnected porous structure, which can accurately control the topology. Finally, the biodegradation rate of it was slow, and the volume loss is about 20% after 4 weeks. However, the corrosion rate of magnesium alloy is relatively fast, accompanied by the concentrated release of a large amount of hydrogen, which makes the human body unable to diffuse and absorb it in time, which will form bubbles on the surface of human skin, affecting the physiological function of tissues around the implant and the restoration treatment of the implant site. Yuan’ s team developed a patented biomedical JDBM material, which has good biocompatibility, good strength and toughness, and low corrosion rate. The process parameters for manufacturing magnesium alloy biomaterials by SLM are currently being developed and are hoped to be applied in the near future [60,104,105,106,107].

In addition to the medical field, magnesium alloys are also widely used in automotive industries. By replacing vehicle components with magnesium-based materials, the vehicle weight can be reduced by 20–70%. Volkswagen began to formally apply magnesium alloy to the automotive industry in 1970. Nowadays, benefitting from the reduced fuel use and carbon dioxide emissions by weight reduction, automobile companies are going to use more magnesium alloys and composites in their products [108]. In the past two decades, the use of magnesium and its alloys in the automotive industry has generally shown an upward trend. Although the cost of parts made by Mg in cars is higher than that of aluminum, it is worthwhile to compensate Mg for its contribution to reducing fuel consumption and carbon dioxide emissions [109]. However, due to its poor ductility and easy corrosion property, magnesium and its alloys cannot fully replace aluminum and its alloys in the application of automotive parts. However, magnesium also has the characteristics of various automotive applications, such as better damping performance than aluminum. Additive manufacturing processes such as SLM are widely used in the lightweight process of automotive parts and the integral molding of complex parts such as support brackets for clutch and brakes, housing for transmission [110]. When the preparation process and other problems are solved, SLM magnesium alloy will also make a difference in the field of automotive weight reduction.

## 6. Opportunities and Challenges

Additive manufacturing technology shows promising potential for development of the future, and it will change the production mode and production site of the manufacturing industry. Selective Laser Melting (SLM) is one of the most attractive metal additive manufacturing (MAM) technologies that allows high precision metal products to be manufactured directly without the need for any molds. Thanks to its high energy input (10–10^3^ J/mm^3^) and ultra-high cooling rate (10^4^–10^6^ K/s), tailored microstructures and tunable complex structures can be obtained from microscale to macroscopic scale combined. In addition, the advantages of reusable recyclable powders, high manufacturing accuracy, and little or no post-processing also expand the possibilities for SLM to manufacture magnesium alloy products.

However, due to the low boiling point (~1091 °C) and the good oxygen affinity of magnesium, there are still many challenges to achieve large-scale applications of SLM in magnesium alloys.

The first is the high manufacturing cost. SLM technology makes magnesium alloy raw materials from spherical metal powders with high purity, narrow particle size distribution and low oxygen content. Due to its easy oxidation and spontaneous combustion problems, magnesium alloy powders are difficult to prepare which leads to additional processing and costs, so the raw material cost of SLM process to manufacture magnesium alloy is much higher than that of traditional technology. At the same time, the current production speed of the SLM process is too slow, resulting in a high depreciation rate of equipment, which further increases the manufacturing cost of SLM. At present, magnesium alloys prepared by SLM are only suitable for high value-added industries such as aerospace, high-end automobiles and biomedicine.

Second, the size of magnesium alloy products manufactured by the SLM process is limited. Due to the limitations of equipment and interlayer resolution, the size of the products manufactured by the current SLM process is usually less than 1 metre, so it is not suitable for SLM technology to prepare large parts.

The third is the structural defects of the magnesium alloy manufactured by SLM. One is that due to the large coefficient of thermal expansion of magnesium alloys and the high cooling rate of the SLM manufacturing process, the manufactured products are prone to defects such as thermal cracks. The other one is that the products produced by additive manufacturing usually leads to anisotropy of the products due to the defect of inter-layer bonding.

## 7. Summary

This paper summarizes the research progress in the field of laser selective melting of magnesium alloys, mainly introduces the influence of magnesium alloy powder and process parameters on laser selective melting (SLM), then discusses the influence on the microstructure, mechanical properties and corrosion resistance of magnesium alloys, and finally prospects the potential application fields of SLM magnesium alloys. Summarized as follows:

(1) High quality powder is the key to improve the manufacturing of magnesium alloy additive. Raw materials are one of the other factors that affect the synthetic properties of SLMed magnesium. Reducing the distribution range of powder particle size, improving the quality of powder, and combining magnesium with other alloy elements can improve product performance. At present, there is no validated commercial magnesium alloy powder material for SLM, the development of new generation high performance magnesium alloy powder for SLM is the key to realize application of the SLMed Magnesium Alloys.
(2) SLM process parameters involve laser power, scanning speed, overlapping distance, layer thickness, scanning angle and others. Adjusting printing parameters can improve tensile strength, hardness and finer microstructure. Due to the low boiling point and the good oxygen affinity of magnesium, violent evaporation splash, large amounts of smoke and dust and recoil pressure on the molten pool will affect the stable melting of the molten pool. During the forming process, appropriate laser energy input and effective gas circulation system can suppress the negative impact of evaporation splashing, thus achieving stable forming quality. It is of great significance to control the defects and increase the window of high-density forming process by adjusting the process parameters. Therefore, more research needs to be done to obtain the optimal printing parameters of various magnesium alloys.
(3) Compared with other materials, Mg has some advantages such as weight, which makes it a potential material to reduce the weight of components in aerospace and automotive industries. In addition, the superior performance of magnesium as a biocompatible and biodegradable material, especially through solid-state laser processing, has attracted more attention to the use of solid-state laser processing of magnesium in the pharmaceutical industry. However, due to the poor corrosion resistance of magnesium alloys, their applications are limited. How to improve the degradation rate of magnesium and its magnesium alloys is the key problem in the application of magnesium alloys.

## Figures and Tables

**Table 1:** Summary of the hardness and microstructural properties of different Mg alloy.

**Materials System**	**Laser Energy Density**	Grain Size (µm)	**Relative Density (Maximum)**	**Hardness**
Pure Mg [49]		2.30–4.87		0.95–0.59 (GPa)
Pure Mg [36]	300 J/mm^3^	1.656–1.671	95.28–96.13	44.75–52.43 Hv
Mg–Ca [64]	600–1200 J/mm^3^	5–30	81.52%	60–68 Hv
Mg–9%Al [46]	7.5–20 J/mm^2^	10–20	82%	~65–85 Hv_25_
AZ61 [65]		5–14	98%	69–93 Hv
AZ91D [47]	83–167 J/mm^3^		99.52%	85–100 Hv
AZ31 [66]	123.81–242.86 J/mm^3^			67–71 Hv
AZ61D [67]		1–3	99.2%	125 Hv
AZ61 + HIP [68]		23.9 ± 6	close to 100%	98.9 ± 5.9 Hv
ZK30 [69]		∼20		80 Hv
WE43 [58]		1.0–1.1		
ZK60 [54]	138.8–416.6 J/mm^3^		94.05%.	~78 Hv
ZK60 [59]	600 J/mm^3^		97.3%	89.2 Hv
Mg–3.4Y–3.6Sm-2.6Zn–0.8Zr [70]		1–3 µm		105 Hv (cross section) 95 Hv (vertical section)
ZK60-Cu [71]		4.5–13.6		80.5 ± 1.9–105.2 ± 2.9
AZ61 [72]	600 J/mm^3^		93.2 ± 2%	90.5 ± 0.9 Hv
AZ61–0.4Mn [72]	600 J/mm^3^	11.4 ± 0.55	91.5 ± 1.8%	95.8 ± 1.2 Hv
AZ61–0.8Sn [72]	600 J/mm^3^		90.3 ± 2.1%	97.2 ± 1.2 Hv
AZ61–0.4Mn-0.8Sn [72]	600 J/mm^3^	4.2 ± 0.42	91.1 ± 1.5%	105 ± 1.4 Hv

**Table 2:** The tensile properties of the as-built and post-processed Mg alloys fabricated by SLM under optimized process parameters.

Alloys	State	YS/MPa	UTS/MPa	EL/%	Ref
ZK60	As-built	137	246	15.5	[83]
	T4	107	224	16.7	
	T6	191	287	14.1	
WE43	As-built	215	251	2.6	[84]
	T6	219	251	4.3	
G10K	As-built	203	271	10.3	[39]
	T5	285	360	2.9	
GZ112K	As-built	332	351	8.6	[85]
	T4	281	311	14.4	
	T6	343	371	4.0	
GZ151K	As-built	345	368	3.0	[86]
	T5	410	428	3.4	
GWZ1031K	As-built	310	347	4.1	[87]
	T5	365	381	0.8	
	T4	255	328	10.3	
	T6	316	400	2.2	

**Table 3:** Summary of corrosion rate of different Mg alloys.

**Materials System**	Tests Solution	Corrosion Rate (mm Year^−1^)	Hydrogen Evolution Rate (ml cm^−2^ h^−1^)
AZ31 [66]	0.9% NaCl solution	0.312	
	3.0% NaCl solution	1.071	
AZ61(80 W) [65]	Simulated body fluid	2.4 (after immersion for 24 h)	
		1.2 (after immersion for 144 h)	
WE43	Hanks’ Balanced Salt Solution	7.04 (as-build)	
		2.11 (grinded batches-SiC4000)	
WE43 [40]	0.1 M NaCl solution	5–7.2	
ZK30 [69]	Simulated body fluid	3.7 0± 0.10	
ZK30–0.6GO [69]		3.38 ± 0.07	
ZK60 [71]	Simulated body fluid	1.01	
ZK60 [59]	Hank’s solution		0.006
Mg–2Mn [93]	Simulated body fluid		0.017
Mg1Zn [94]	C-simulated body fluid		0.17

## Data Availability

Not applicable.
